# A Model for Estimating Tactile Sensation by Machine Learning Based on Vibration Information Obtained while Touching an Object

**DOI:** 10.3390/s21237772

**Published:** 2021-11-23

**Authors:** Fumiya Ito, Kenjiro Takemura

**Affiliations:** 1 Graduate School of Science for Open and Environmental Systems, Keio University, Yokohama 223-8522, Japan; f.i_0110.apple@keio.jp; 2 Department of Mechanical Engineering, Keio University, Yokohama 223-8522, Japan

**Keywords:** tactile sensor, tactile estimation, machine learning, vibration, feature extraction, sensory evaluation

## Abstract

The tactile sensation is an important indicator of the added value of a product, and it is thus important to be able to evaluate this sensation quantitatively. Sensory evaluation is generally used to quantitatively evaluate the tactile sensation of an object. However, statistical evaluation of the tactile sensation requires many participants and is, thus, time-consuming and costly. Therefore, tactile sensing technology, as opposed to sensory evaluation, is attracting attention. In establishing tactile sensing technology, it is necessary to estimate the tactile sensation of an object from information obtained by a tactile sensor. In this research, we developed a tactile sensor made of two-layer silicone rubber with two strain gauges in each layer and obtained vibration information as the sensor traced an object. We then extracted features from the vibration information using deep autoencoders, following the nature of feature extraction by neural firing due to vibrations perceived within human fingers. We also conducted sensory evaluation to obtain tactile scores for different words from participants. We finally developed a tactile sensation estimation model for each of the seven samples and evaluated the accuracy of estimating the tactile sensation of unknown samples. We demonstrated that the developed model can properly estimate the tactile sensation for at least four of the seven samples.

## 1. Introduction

Quantitative evaluation of tactile sensation is important in industry because tactile sensation is a characteristic of products that relates directly to product value, especially for consumer products [1,2,3,4]. Sensory evaluation is generally conducted to quantify tactile sensation but requires an extremely large number of participants to ensure reliability, resulting in high financial and time costs. There is thus strong demand for the establishment of tactile sensing technology.

A number of studies have endeavored to establish tactile sensing technology [5,6,7,8,9,10,11,12,13,14,15,16,17]. Chen et al. [5] recorded vibration data when an artificial finger ran over a cloth sample. The vibration was detected from a sound wave traveling through a conductive liquid in the artificial finger. They found that the peak average, power, Shannon entropy calculated from the vibration data, and friction coefficient characterize the tactile sensation of a cloth sample. Asaga et al. [6] proposed a method of evaluating the tactile sensation of fabric, leather, plastic, and paper samples based on the human mechanism of tactile perception. They measured the output waveform when the sample surface was touched with a piezoelectric element and estimated the firing status of each mechanoreceptor by comparing the vibration waveform and firing threshold of each mechanoreceptor in the frequency domain. In addition, they compared the firing status with the results of the sensory evaluation of each sample and concluded that they could estimate the tactile sensation of softness and roughness from the quantitatively estimated firing status of mechanoreceptors. A similar approach has been applied to different samples [7,8]. Kerzel et al. [9] used an optical force sensor mounted on a robot arm to measure vibration generated by friction and texture. They then extracted the relevant spectral features from the obtained information and used a multi-neural network to classify the materials of 32 different samples. Saga et al. [10] traced the surface of an object using either a finger or a pen equipped with a wireless accelerometer and classified 15 kinds of sample material using the collected tactile information.

Previous research on tactile sensing technology can be divided into two main categories: (a) the research uses physical properties such as friction and roughness, as well as other information acquired by sensors, to understand the relationship between the tactile and physical properties of a sample, or (b) the research on physical analysis of samples, such as material classification and force prediction, based on information acquired by sensors. However, research of category (a) does not consider the evaluation of the tactile sensation of an unknown sample and research of category (b) only analyzes the physical properties of samples and does not evaluate or explain the tactile sensation. In particular, there has been little research on an effective feature extraction strategy for physical measurements. As a human has an effective feature extraction function in the form of mechanoreceptors [18,19], an effective feature extractor should be designed for an artificial tactile sensor.

As for feature extraction, various research has been conducted. Neural networks have recently been applied to a variety of nonlinear problems [20,21,22], among which the deep autoencoder approach has been widely used in dimensionality reduction and feature extraction [23]. Deep autoencoder has been proven to be an effective way to learn and describe latent codes that reflect meaningful variations in data with encoder and decoder [23,24]. Deep autoencoder has also been applied to many vibration-related problems, such as vibration-based feature extraction and fault detection [25,26,27]. Therefore, it must be reasonable to introduce deep autoencoder to extract features from the vibration information acquired by a tactile sensor. This research, therefore, first fabricates a tactile sensor capable of collecting vibration information and introduces the feature extraction function from the vibration information by deep autoencoder. Then, the present research develops a machine learning model that estimates the tactile sensation of an unknown sample by modeling the relationship between the extracted features and human tactile sensation scores collected with sensory evaluation tests.

The paper is organized as follows: Section 2 presents details of the research methodology and describes the tactile sensor developed in this study, followed by the structure of neural networks. Section 3 and Section 4 present the experimental results and discussions, respectively. Then, the conclusion of this study is given in Section 5.

## 2. Materials and Methods

### 2.1. Strategy for Tactile Model Development

**Figure 1:**
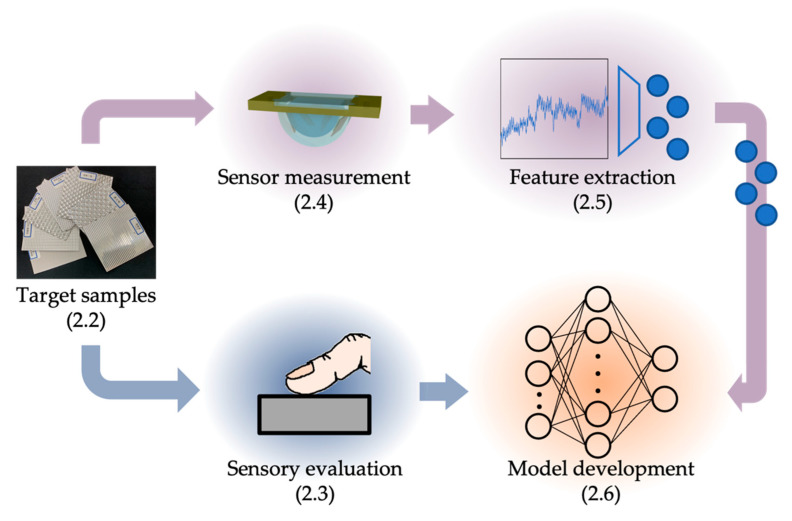
Strategy for developing a tactile estimation model. The number in parenthesis for each item refers to the section in which the item is described, (Section 2.2) target samples, (Section 2.3) sensory evaluation of samples, (Section 2.4) tactile sensing system and experimental conditions, (Section 2.5) feature extraction for the data acquired by the autoencoder, and (Section 2.6) establishment of a tactile estimation model through machine learning.

An approach to developing a tactile estimation model is shown in Figure 1. We first conducted a sensory evaluation of human subjects. In parallel, we fabricated a tactile sensor and acquired vibration information while the tactile sensor ran over each sample. Features of vibration information were then extracted by deep autoencoders. Finally, a model for estimating tactile sensation was developed by modeling the relationship between the features of vibration as input and the tactile evaluation score as output.

### 2.2. Target Samples

**Figure 2:**
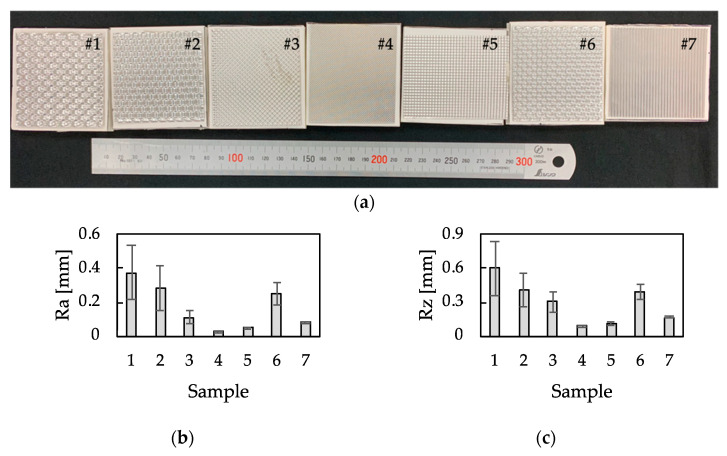
Information about seven aluminum samples. All samples are embossed. (**a**) Images of aluminum test samples. The sample number is given above each sample. (**b**) The arithmetic mean roughness (Ra), (**c**) the maximum height (Rz) (n=10, mean±SD).

Seven aluminum plate samples, shown in Figure 2a, were used as test samples. The surface of each sample was embossed to provide a different tactile sensation, and the arithmetic mean roughness (Ra) and the maximum height (Rz) of seven samples are shown in Figure 2b,c, respectively. A cardboard plate was glued onto the back side of each sample to prevent a possible different friction feeling when it was employed in sensory evaluation experiments. In order to demonstrate that the developed system can detect the difference in tactile sensation, which is due to the difference in shape, we employed the samples with different surfaces with the same material.

### 2.3. Sensory Evaluation of Samples

A sensory evaluation was performed by test participants to provide tactile scores for the seven aluminum samples. The sensory evaluation was conducted at a temperature of 23.0 ± 1.6 °C and relative humidity of 30.9% ± 1.6% by 14 healthy adults (12 males and 2 females), aged 23.3 ± 1.0 years (ranging 21–25 years). We employed a semantic differential method with a seven-step unipolar scale. Following the previous research [8], we employed the unipolar scale to avoid translation problems between opposite adjectives [28]. Moreover, the scale is only defined at the endpoints to prevent varying interpretations of verbal anchors and unevenness between anchors [29]. Nine Japanese adjectives (Table 1), which were determined in a preliminary experiment, were used as evaluation words. In the preliminary experiment, 28 Japanese adjectives obtained by brainstorming of tactile researchers were prepared, and we asked six participants (aged 23.2 ± 1.5 years, ranging 21–25 years) whether it was possible to evaluate the samples using each of 28 Japanese adjectives. Subsequently, the adjectives for which more than 80% (at least five out of six) of the participants found suitable for evaluation were considered suitable for the evaluation of the aluminum samples. On the basis of this criterion, 9 of the 28 adjectives were adopted as evaluation words. During the preliminary experiment and the sensory evaluation, each sample was put in a box so that visual information was excluded. The participants were requested to actively trace his/her finger on the samples in the horizontal direction. In advance to the evaluation, the participants touched all the samples to understand the population of samples. Additionally, in order to avoid the possible order effect in the sensory evaluation, the participants were free to touch the samples in random order. In addition, the participants could touch all the samples while scoring an evaluation word. A 30 s interval was placed after the participant had evaluated two evaluation words in order to reduce the fatigue of the participant and to reduce possible heat transfer from the finger to the aluminum plate samples. The test protocol was approved in advance by The Bioethics Board of the Faculty of Science and Technology, Keio University. The participants were provided a thorough explanation of the evaluation methods and then signed an informed consent form before participating in the study.

### 2.4. Tactile Sensing System and Experimental Conditions

**Figure 3:**
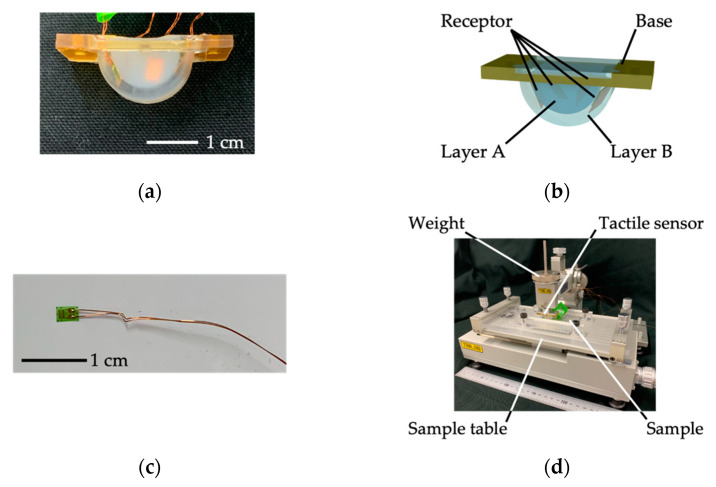
Tactile sensing system with the developed tactile sensor: (**a**) photograph of the fabricated tactile sensor, (**b**) overview of the tactile sensor, (**c**) photograph of the strain gauge, and (**d**) overall view of the sensing system. The image in (**d**) shows the tactile sensor running over a sample as a result of the sliding of the sample table.

We developed a tactile sensing system that detects vibration when a tactile sensor runs over a sample. Figure 3a,b, respectively, show the actual tactile sensor and its structure. As shown in Figure 3b, the tactile sensor comprises two layers of silicone rubber with different hardness; layer A is made of a silicone rubber (KE-1316, Shin-Etsu Chemical Co., Ltd., Tokyo, Japan) whereas layer B is made of another silicone rubber (SYLGARDTM 184, The Dow Chemical Company, Midland, MI, USA). The hardness value (as measured by a type-A durometer) is 23 and 43 for layers A and B, respectively [30,31], so the silicone rubber for layer B is harder than that for layer A. Note that the dermis inside the human finger is harder than the subcutaneous tissue [32]. Each layer contains two strain gauges, shown in Figure 3c (KFGS-03-120-C1-23, Kyowa Electronic Instruments Co., Ltd., Tokyo, Japan), such that a total of four strain gauges measure the vibration generated inside the tactile sensor when the tactile sensor runs over a sample surface. Each of the four strain gauges is glued onto a phosphor bronze plate with an adhesive (CC-33A, Kyowa Electronic Instruments Co., Ltd., Tokyo, Japan), and we refer to such a strain gauge with the phosphor plate as a receptor. There are thus two receptors in each layer, making it possible to collect four sets of vibration information in a single measurement. Furthermore, since the two receptors in each layer are placed in random positions and directions, a variety of information can be collected in a single measurement. The two receptors in layer A are denoted A1 and A2 and the two receptors in layer B are denoted B1 and B2. The output of the strain gauges is acquired through four dynamic strain amplifiers (DPM-911B, DPM-913B, DPM-913C, Kyowa Electronic Instruments Co., Ltd., Tokyo, Japan). The specific characteristics for dynamic strain amplifiers are shown in Table S1. Figure 3d shows the overall appearance of the system, in which the tactile sensor mentioned above is attached to the arm of the static/dynamic friction-measuring instrument (TL201Ts, Trinity-lab.INC., Tokyo, Japan). The specific characteristics for the static/dynamic friction measuring instrument are shown in Table S2. The load pressing down on the sample during the experiment can be adjusted using a weight. As the sample table of the TL201Ts static/dynamic friction measuring instrument moves horizontally, the sensor runs over a sample. Vibration generated by the relative slippage between the sensor and sample is measured through the four receptors. The measurement conditions in the present study were a running speed of the tactile sensor of 20 mm/s, a running distance of 50 mm, and a weight (corresponding to the sample pressing force) of 0.98 N. Measurements were acquired for a period of 2 s (from 0.25 to 2.25 s) from a tracing of about 2.5 s.

### 2.5. Feature Extraction for the Data Acquired by the Autoencoder

There are four types of mechanoreceptors in the glabrous skin of a human, namely Meissner corpuscles (FA I), Pacinian corpuscles (FA II), Merkel disks (SA I), and Ruffini endings (SA II) [18,33,34]. These mechanoreceptors constitute mechanoreceptive units with corresponding neurons. The mechanoreceptive units respond to mechanical stimuli induced by vibration inputs and fire nerve impulses to neurons. Considering this human feature extraction function, an autoencoder [35] with a deep neural network is used to implement a feature extraction function in the tactile sensing system.

**Figure 4:**
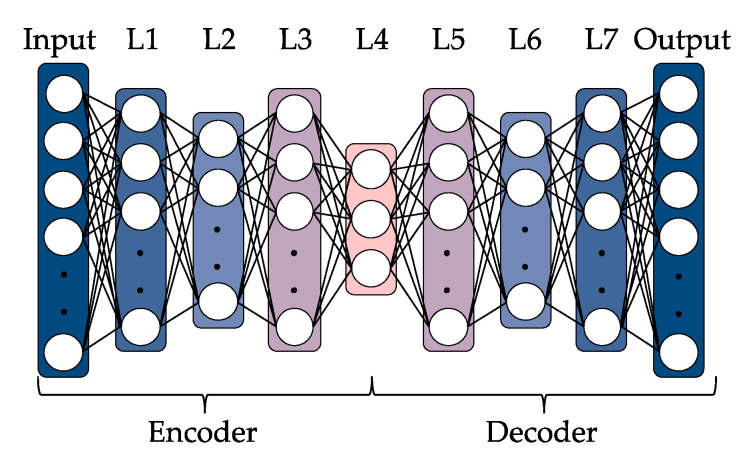
Diagram of the developed deep autoencoder. L1–L7 are the intermediate layers and L4 is the feature extraction layer. The numbers of neurons are the same for L1 and L7, L2 and L6, and L3 and L5. The dimension of features, which is the number of neurons in L4, is three and the numbers of neurons in L1, L2, L3, L5, L6, and L7 are optimized in the range of 5 to 999 by Optuna.

An autoencoder is a kind of neural network used for dimensional compression. The simplest autoencoder has three layers, namely an input layer, intermediate layer, and output layer. The numbers of neurons in the input and output layers are set equal, and the same data are used as the input and the target output during training. The number of neurons in the intermediate layer is set to be smaller than that in the input and output layers. The layer that performs compression (from the input layer to the intermediate layer) is called the encoder, and the layer that performs restoration (from the intermediate layer to the output layer) is called the decoder. By learning the parameters that minimize the difference between the input and target output, the output values of the neurons in the intermediate layer can be used as dimensionally compressed features [35]. The deep autoencoder shown in Figure 4 is an application of the autoencoder. It has been shown that a neural network with more than one intermediate layer and a sufficiently large number of neurons in the intermediate layer can approximate arbitrary functions [36,37]. This approximation allows for the use of a deep autoencoder to develop models that can be used for more diverse expressions compared to a simple autoencoder with a single intermediate layer.

**Figure 5:**
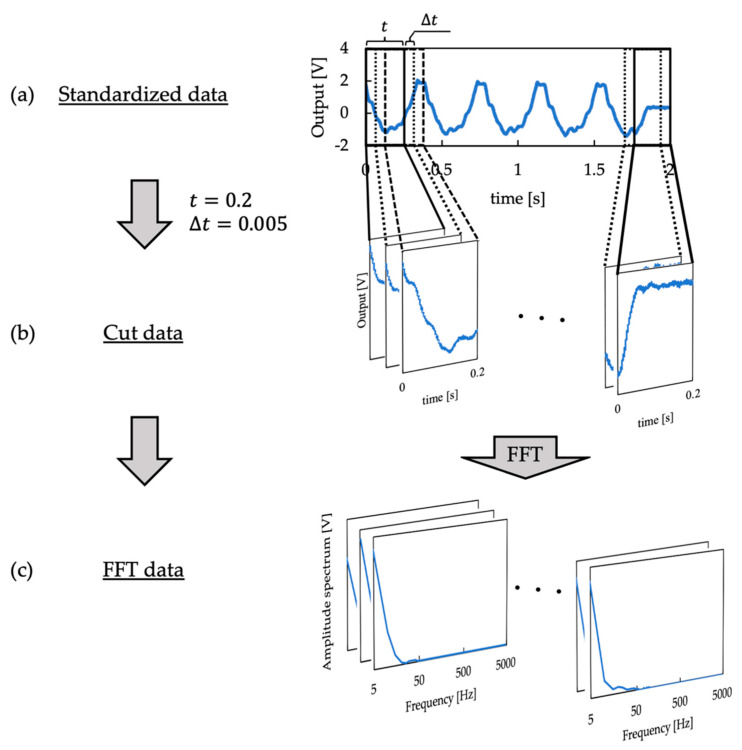
Procedure of generating the input vibration data for deep autoencoders.

Vibration input to the autoencoder was generated as shown in Figure 5. First, the measurement results of vibration obtained in Section 2.4 were standardized to have a mean of zero and variance of 1. The equation of standardization for a certain data point $d_{i}$ is written as

(1) $$d_{i}^{\prime }=\frac{d_{i}-d_{mean}}{d_{std}},$$

where $d_{mean}$ is the average and $d_{std}$ is the standard deviation of all data points (corresponding to (a) in Figure 5). Next, from vibration information having a total time length of 2 s, the information for a period of 0.2 s was slid by 0.005 s, and 361 pieces of vibration information for a period of 0.2 s were extracted per measurement (corresponding to (a) to (b) in Figure 5). In other words, 25,270 pieces of vibration information for each period of 0.2 s were extracted for each of the four receptors, A1, A2, B1, and B2. A fast Fourier transformation (FFT) was then performed on the vibration information for each period of 0.2 s to obtain 1000-dimensional information in the frequency range of 5 to 5000 Hz in 5 Hz increments (corresponding to (b) to (c) in Figure 5). The FFT was performed using the Python library NumPy [38] with 2000 data points and a Hamming window as the window function.

In this research, we used a hyperparameter optimization algorithm to develop deep autoencoders. In neural networks, there are weights and biases that are optimized by learning and other parameters, such as the learning rate and number of epochs, which are not optimized by learning. The parameters that are not optimized by learning are called hyperparameters. In this study, we used Optuna [39], which adjusts hyperparameters using a Tree-structured Parzen Estimator [40].

We developed deep autoencoders for feature extraction using the input information and hyperparameter optimization algorithm described above. We used Keras [41], a Python framework for deep learning, to develop the deep autoencoders. There were 25,270 datasets of 1000-dimensional data as mentioned above. To evaluate the generalization ability of the deep autoencoder, we first used 80% of a dataset for training and the remaining 20% for testing, and we did not use any test data when optimizing the hyperparameters and training the deep autoencoder. Then, using the training dataset, we developed a deep autoencoder with nine layers and the three-dimensional extraction of features, as shown in Figure 4. The preset parameters are given in Table 2. In addition, the numbers of neurons in the intermediate layers L1–L3 and L5–L7 shown in Figure 4 were set at 5 to 999 and optimized by Optuna. The number of neurons in each intermediate layer optimized by Optuna is given in Table 3. Note that in hyperparameter optimization, 75% of the training dataset was used for training and 25% for verification.

After the optimization of hyperparameters, the training dataset was used to train the deep autoencoder. The training was performed using the cross-validation method with k=8, and the number of training epochs at each evaluation was 200. On the above basis, vibration information with 1000 dimensions was compressed to three dimensions. In other words, three features were extracted for a single receptor. This feature extraction procedure was performed for all four receptors, and 12-dimensional features (four receptors and three features for each receptor) were thus extracted.

### 2.6. Establishment of a Tactile Estimation Model through Machine Learning

A model for estimating tactile sensation was constructed by modeling the relationship between the 12-dimensional feature of vibration information obtained in Section 2.4 and Section 2.5 as input and the nine-dimensional tactile evaluation of aluminum plates (scores of nine evaluation words) as output. There were 3610 data for the features extracted from the vibration information in Section 2.5, whereas there were 14 tactile evaluation data obtained in the sensory evaluation in Section 2.3, and the data for sensory evaluation were replicated until there were 3610 data per sample such that the numbers of data were made equal. To evaluate the generalization performance of the tactile sensation estimation model for unknown data, we trained the model for data of six of the seven aluminum plate samples and used the developed model to estimate the tactile evaluation score for the remaining sample. A total of seven models were constructed such that each of the seven samples was used as the unknown sample, and the generalization performance of each model was evaluated. In the evaluation, the results of the tactile sensation estimation using each model were compared with the tactile evaluation score given by the human participants in sensory evaluation.

An all-coupled neural network was used for model development to consider the nonlinearity of the human tactile perception mechanism. As in the case of feature extraction, the optimization of hyperparameters by Optuna was performed for the number of intermediate layers (one to four layers) and the number of neurons in each intermediate layer (1–500 neurons). The parameters predetermined for optimization and the number of optimized intermediate layers are given in Table 4 and Table 5, respectively. For all models, the optimized number of intermediate layers was four. Note that each intermediate layer is arranged in the order of input–L1–L2–L3–L4-output in Table 5.

The optimized hyperparameters were used to train the tactile sensation estimation model for each of the seven samples. Training was performed using the cross-validation method with k=10, and the number of epochs in each evaluation was 200. After confirming that there was no overtraining in any evaluation, we evaluated the tactile sensation of each sample to be estimated using the seven models developed.

## 3. Results

### 3.1. Sensory Evaluation Results

**Figure 6:**
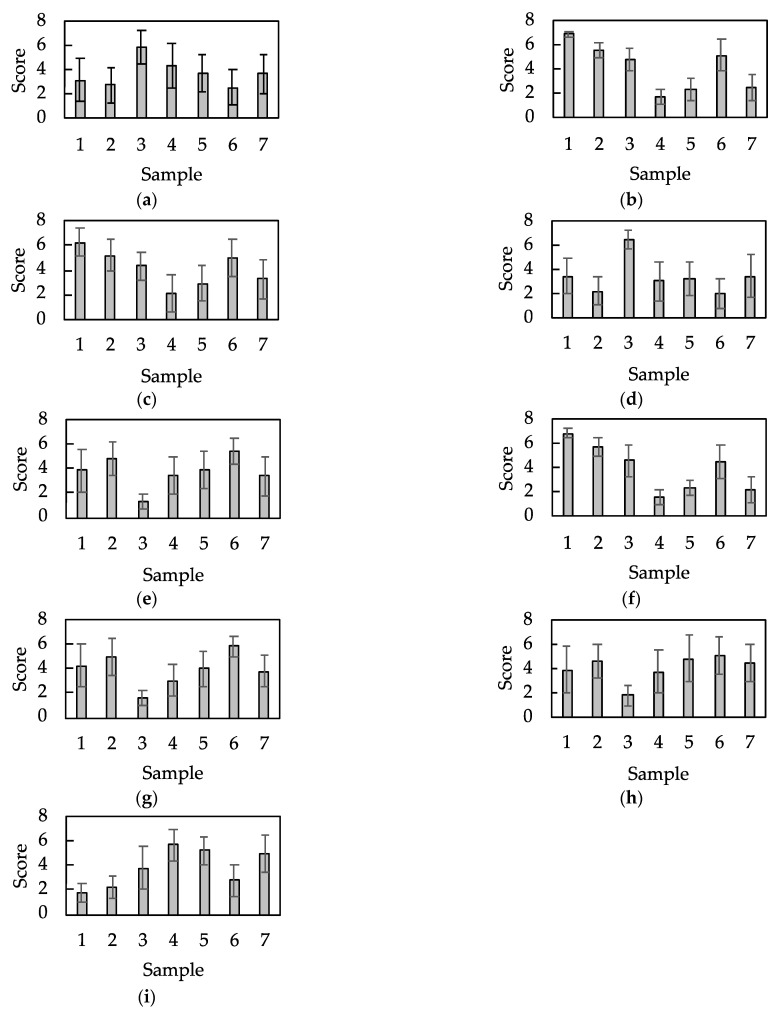
Results of the sensory evaluation of the seven aluminum samples. A semantic differential method with a seven-step unipolar scale was employed for sensory evaluation. (**a**–**i**) represent the words, (**a**) rough, (**b**) uneven, (**c**) coarse, (**d**) prickly, (**e**) smooth, (**f**) rugged, (**g**) slippery, (**h**) sleek, and (**i**) dry (n=14, mean±SD).

The results of the sensory evaluation are shown in Figure 6. Each graph presents the mean score and standard deviation for each evaluation term used. The figure shows that there are obvious differences in the tactile sensation among the samples.

### 3.2. Feature Extraction from Acquired Vibration Data

**Figure 7:**
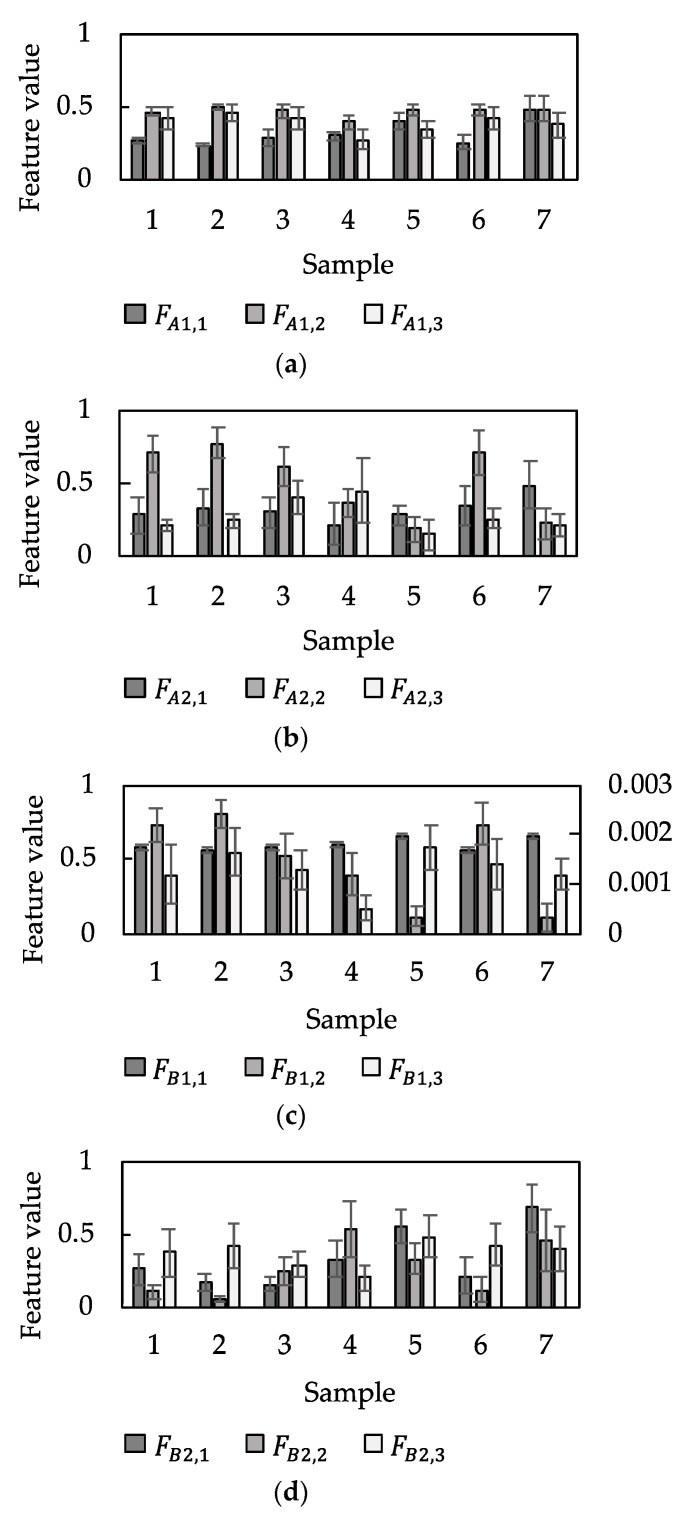
Results of feature extraction. Graphs show the feature values for (**a**) A1, (**b**) A2, (**c**) B1, and (**d**) B2 (n=3610, mean±SD). The vertical axis on the right in (**c**) gives the value of $F_{B1,1}$.

After the deep autoencoder was trained for each receptor, the outputs of the three neurons in the feature extraction layer (L4 in Figure 4) were extracted and used as the feature values in the following tactile estimation. Figure 7 shows the 12 extracted features for the four receptors against the seven samples. The feature values are well distributed. Table 6 gives the generalization error when the trained model was applied to the test data. The small error in feature extraction given in the table is small enough compared with the input/output data being standardized with a mean of zero and variance of 1.

### 3.3. Tactile Estimation Models Developed through Machine Learning

**Figure 8:**
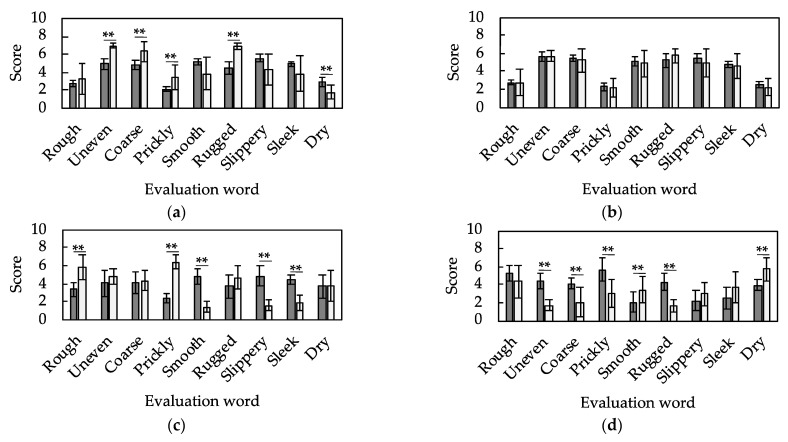

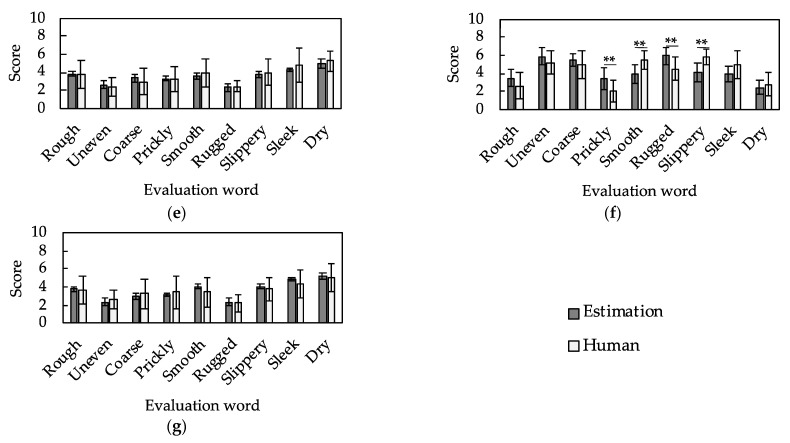
Comparison of the mean and standard deviation of the tactile scores estimated by the estimation model (gray, n=3610, mean±SD) and the evaluation values obtained in the sensory evaluations by human participants (white, n=14, mean±SD) for the nine evaluation words in each sample. Each panel is the result for a model that estimates the tactile sensation of (**a**) sample 1, (**b**) sample 2, (**c**) sample 3, (**d**) sample 4, (**e**) sample 5, (**f**) sample 6, and (**g**) sample 7 (** p<0.01).

The optimized hyperparameters were used to develop the tactile estimation models. The vibration features of the unknown samples were input to each of the developed models, and the mean squared errors, which means differences between the output tactile sensation estimates and the tactile sensation evaluations obtained in human sensory evaluation, are compared in Table 7. Figure 8 compares the estimated tactile scores of the nine evaluation words for each sample and the evaluation scores obtained in the sensory evaluations performed by the human participants. In the figure, samples with p<0.01 for the majority of the nine evaluation words (samples 1, 3, and 4) are considered to have not been properly estimated for tactile sensation, whereas the remaining samples (samples 2, 5, 6, and 7) are considered to have been properly estimated. The samples that were not properly estimated correspond to the samples with mean squared errors larger than 4 in Table 7. The generalization error in training is important; however, tactile sensation was successfully estimated for four of the seven samples.

## 4. Discussion

As described in Section 3.3, there are two groups of samples. That is, the tactile sensations of samples 2, 5, 6, and 7 can be properly estimated by the developed system, whereas those of samples 1, 3, and 4 cannot. We discuss here two aspects of the differences between the two groups, namely the different characteristics of each sample and the learning status of the neural network, to clarify what type of sample is suited to our tactile estimation system. To understand the difference in samples, we performed cluster analyses based on the evaluation scores from sensory evaluation and on features extracted from vibration data obtained with the tactile sensor. In the cluster analysis, we employed the Ward method, which is an agglomerative hierarchical method [43,44]. The calculation was conducted with Scipy, a Python library [45].

**Figure 9:**
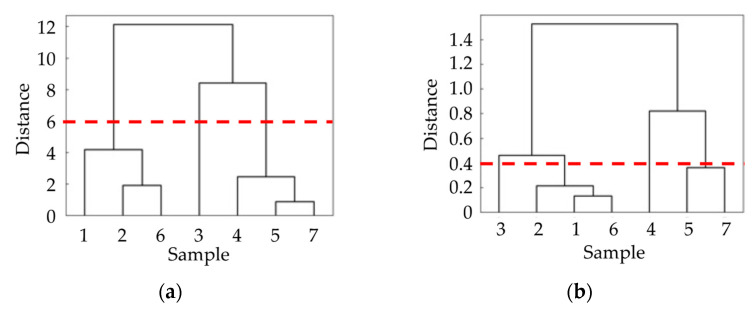
Results of cluster analysis: (**a**) cluster analysis of the samples based on the evaluation words obtained in the sensory evaluation and (**b**) cluster analysis of the features of the vibration information.

Figure 9a shows the results of cluster analysis based on the sensory evaluation scores, whereas Figure 9b shows the results of cluster analysis based on the extracted features of vibration information. Figure 9a presents the similarity of the tactile sensations that the participants actually feel, whereas Figure 9b presents the similarity of the information acquired by the tactile sensor. It is seen that the tactile sensation (human) and vibration feature (tactile sensor) are similar for samples 5 and 7. Figure 8e,g show that the tactile sensations of these samples are well estimated by the developed system. Additionally, samples 2 and 6 are close in Figure 9a,b, and their tactile estimations are successfully conducted. Note that the term “successfully” here means that the majority of evaluation words are statistically well estimated. Meanwhile, samples 1, 3 and 4 are distant from the other samples in Figure 9. Sample 1 is close to sample 6 in terms of the extracted vibration feature (Figure 9b) but not in terms of the evaluation score (Figure 9a). This implies that the samples that have almost the same similarity pair in Figure 9a,b are suitable for the developed tactile estimation system. The sample information (Figure 2) and sensory evaluation scores (Figure 6) reveal that a successful estimation (samples 2, 5, 6, and 7) was made for the samples with a relatively flat surface, wide unevenness width, and rugged texture. The estimation failed for sample 1 having deep unevenness (Figure 6b, uneven), sample 3 having a narrow unevenness width (Figure 6d, prickly), and sample 4 having a dot-like texture (Figure 6f, rugged). From the viewpoint of learning, these samples are isolated in terms of texture among the prepared sample group, and the appropriate learning of these samples was thus difficult. However, as described above, the tactile estimation of samples having similar texture in the prepared sample group was successfully conducted.

The above discussion suggests that our strategy can contribute to the establishment of an effective and quantitative tactile estimation model, provided that an appropriate target sample group is prepared. In addition, by having demonstrated the efficacy of using deep autoencoders to extract feature quantities in tactile sensing, it was shown that the system can be extended to use other parameters such as temperature for tactile estimation in future work. Any physically developed tactile sensor system can have its own feature extractor through the use of a deep autoencoder, just as humans have acquired mechanoreceptive units, namely the feature extractors in our glabrous skin.

## 5. Conclusions

The tactile sensation is an important characteristic of a consumer product and is strongly related to product evaluation. The estimation of tactile sensation is thus a major topic in the field of sensing technology. We developed in this study a tactile sensing technology by developing a tactile sensor, a feature extractor of vibration data, and an estimation model of tactile sensation from sensor data through machine learning. We first fabricated the tactile sensor with two silicone layers with two strain gauges in each layer capable of measuring vibration data when the sensor runs over a sample. In addition, instead of using the raw vibration data obtained by the sensor, we extracted the features from the vibration data using a deep autoencoder, considering that feature extraction is performed at mechanoreceptors in human fingers. Sensory evaluation was also conducted to obtain human tactile evaluation scores. We finally modeled the relationship between the extracted feature values of the vibration data recorded by the sensor as input and the human tactile evaluation scores obtained in the sensory evaluation as output using an all-coupled neural network. We thus demonstrated that the tactile sensations of four of seven samples could be successfully estimated. A detailed discussion suggested that the success of the estimation depends on the prepared sample group.

Our method allows us to understand the tactile sensation of a product from a simple measurement. It can be applied to product development processes to reduce time and economic costs. In future studies, the sensor’s design may be optimized to make it possible to estimate the tactile sensation of samples with even more diverse shapes. Furthermore, the generality of this study can be enhanced by applying the developed system to samples with different materials.

## Figures and Tables

**Table 1:** Words used in the sensory evaluation test (terms in brackets are in Japanese).

Evaluation Words (Japanese)		
Rough (Zarazara-suru)	Uneven (Dekoboko-suru)	Coarse (Kime-no-arai)
Prickle (Chikuchiku-suru)	Smooth (Namerakana)	Rugged (Gotsugotsu-suru)
Slippery (Tsurutsuru-suru)	Sleek (Subesube-suru)	Dry (Sarasara-suru)

**Table 2:** Preset parameters for the deep autoencoders.

The number of neurons of the input layer	1000
The number of neurons of the output layer	1000
The number of neurons of the feature extraction layer (L4)	3
The number of intermediate layers of encoder and decoder	3
Weight optimization algorithm	Adam [42] $\beta_{1}:0.9$ $\beta_{2}:0.999$ $\epsilon :10^{-7}$ α=0.001
Activation function	Encoder: sigmoid Decoder: ReLU
Loss function	Mean squared error
Batch size	128
Trial number of Optuna	200
Epochs	200

**Table 3:** Optimized number of neurons in intermediate layers for the four autoencoders.

	A1	A2	B1	B2
L1 and L7	606	505	957	951
L2 and L6	96	306	43	278
L3 and L5	37	154	324	266

**Table 4:** Preset parameters for the tactile sensation estimation model.

The number of neurons of the input layer	12
The number of neurons of the output layer	9
The number of neurons of the feature extraction layer (L4)	21,660
Weight optimization algorithm	Adam $\beta_{1}:0.9$ $\beta_{2}:0.999$ $\epsilon :10^{-7}$ α=0.001
Activation function of the output layer	Linear
Activation function other than the output layer	sigmoid
The ration of train data and verification data	4:1
Loss function	Mean squared error
Batch size	128
Trial number of Optuna	100
Epochs	200

**Table 5:** Optimized number of neurons in intermediate layers for each tactile sensation estimation model.

Model	L1	L2	L3	L4
Sample 1	449	442	155	150
Sample 2	484	498	207	50
Sample 3	399	412	408	402
Sample 4	439	447	236	21
Sample 5	474	446	157	411
Sample 6	266	287	244	35
Sample 7	492	481	320	116

**Table 6:** Error in applying the trained deep autoencoder to the test data.

Receptor	$\mathrm{Generalization}\;\mathrm{Error}\;[\times 10^{-5}\;V^{2}]$
A1	2.20
A2	2.93
B1	4.09
B2	3.11

**Table 7:** Error in applying the trained tactile sensation model to unknown data.

Model	Generalization Error [-]
Sample 1	4.05
Sample 2	1.75
Sample 3	8.33
Sample 4	6.68
Sample 5	2.08
Sample 6	3.99
Sample 7	2.38

## Data Availability

The data presented in this study are available on request from the corresponding author.

## Supplementary Materials

The following are available online at https://www.mdpi.com/article/10.3390/s21237772/s1, Table S1: Specific characteristics for dynamic strain amplifiers, Table S2: Specific characteristics for the static/dynamic friction measuring instrument.

## References

[1] Grohmann B., Spangenberg E.R., Sprott D.E. (2007). The influence of tactile input on the evaluation of retail product offerings. J. Retail..

[2] Yanagisawa H., Fukuda S. (2011). Kansei Quality in Product Design. Emotional Engineering Service Development.

[3] Jansson-Boyd C.V. (2011). Touch matters: Exploring the relationship between consumption and tactile interaction. Soc. Semiot..

[4] Atefeh Y., Nancy S. (2013). Can consumers forgo the need to touch products? An investigation of nonhaptic situational factors in an online context. Psychol. Mark..

[5] Chen S., Ge S., Tang W., Zhang J., Chen N. (2015). Tactile perception of fabrics with an artificial finger compared to human sensing. Text. Res. J..

[6] Asaga E., Takemura K., Maeno T., Ban A., Toriumi M. (2013). Tactile evaluation based on human tactile perception mechanism. Sens. Actuators A Phys..

[7] Nobuyama L., Kurashina Y., Kawauchi K., Matsui K., Takemura K. (2018). Tactile Estimation of Molded Plastic Plates Based on the Estimated Impulse Responses of Mechanoreceptive Units. Sensors.

[8] Hashim I.H.M., Kumamoto S., Takemura K., Maeno T., Okuda S., Mori Y. (2017). Tactile Evaluation Feedback System for Multi-Layered Structure Inspired by Human Tactile Perception Mechanism. Sensors.

[9] Kerzel M., Ali M., Ng H.G., Wermter S. Haptic material classification with a multi-channel neural network. Proceedings of the 2017 International Joint Conference on Neural Networks (IJCNN).

[10] Saga S., Agatsuma S., Vasilache S., Takahashi S. (2020). Machine learning-based classification and generation of vibrotactile information. Int. J. Adv. Netw. Svcs..

[11] Li G., Liu S., Wang L., Zhu R. (2020). Skin-inspired quadruple tactile sensors integrated on a robot hand enable object recognition. Sci. Robot..

[12] Gandarias J.M., Gómez-De-Gabriel J.M., García-Cerezo A. Human and object recognition with a high-resolution tactile sensor. Proceedings of the 2017 IEEE SENSORS.

[13] Massari L., Schena E., Massaroni C., Saccomandi P., Menciassi A., Sinibaldi E., Oddo C.M. (2020). A Machine-Learning-Based Approach to Solve Both Contact Location and Force in Soft Material Tactile Sensors. Soft Robot..

[14] Huang S., Wu H. (2021). Texture Recognition Based on Perception Data from a Bionic Tactile Sensor. Sensors.

[15] Chun S., Hwang I., Son W., Chang J.-H., Park W. (2018). Recognition, classification, and prediction of the tactile sense. Nanoscale.

[16] Qin L., Yi Z., Zhang Y. (2017). Enhanced surface roughness discrimination with optimized features from bio-inspired tactile sensor. Sens. Actuators A Phys..

[17] Hosoda K., Tada Y., Asada M. (2006). Anthropomorphic robotic soft fingertip with randomly distributed receptors. Robot. Auton. Syst..

[18] Johansson R.S., Landstrom U., Lundstrom R. (1982). Responses of mechanoreceptive afferent units in the glabrous skin of the human hand to vibration. Brain Res..

[19] Bolanowski S.J., Gescheider G.A., Verrillo R.T., Checkosky C.M. (1988). Four channels mediate the mechanical aspects of touch. J. Acoust. Soc. Am..

[20] Raissi M., Perdikaris P., Karniadakis G. (2019). Physics-informed neural networks: A deep learning framework for solving forward and inverse problems involving nonlinear partial differential equations. J. Comput. Phys..

[21] Anitescu C., Atroshchenko E., Alajlan N., Rabczuk T. (2019). Artificial Neural Network Methods for the Solution of Second Order Boundary Value Problems. Comput. Mater. Contin..

[22] Weinan E., Han J., Jentzen A. (2017). Deep Learning-Based Numerical Methods for High-Dimensional Parabolic Partial Differential Equations and Backward Stochastic Differential Equations. Commun. Math. Stat..

[23] Zhuang X., Guo H., Alajlan N., Zhu H., Rabczuk T. (2021). Deep autoencoder based energy method for the bending, vibration, and buckling analysis of Kirchhoff plates with transfer learning. Eur. J. Mech.-A/Solids.

[24] Snoek J., Adams R.P., Larochelle H. (2012). Nonparametric guidance of autoencoder representations using label information. J. Mach. Learn. Res..

[25] Qu Y., He M., Deutsch J., He D. (2017). Detection of Pitting in Gears Using a Deep Sparse Autoencoder. Appl. Sci..

[26] Shang Z., Sun L., Xia Y., Zhang W. (2021). Vibration-based damage detection for bridges by deep convolutional denoising autoencoder. Struct. Health Monit..

[27] Shao H., Jiang H., Zhao H., Wang F. (2017). A novel deep autoencoder feature learning method for rotating machinery fault diagnosis. Mech. Syst. Signal Process..

[28] Tuorila H., Huotilainen A., Lähteenmäki L., Ollila S., Tuomi-Nurmi S., Urala N. (2008). Comparison of affective rating scales and their relationship to variables reflecting food consumption. Food Qual. Prefer..

[29] Cantin I., Dubé L.L. (1999). Attitudinal Moderation of Correlation between Food Liking and Consumption. Appetite.

[30] Liquid Silicone Rubber for Moldmaking. https://www.shinetsusilicone-global.com/catalog/pdf/mold_silicone_e.pdf.

[31] SYLGARD™ 184 Silicone Elastomer Kit Technical Data Sheet. https://www.dow.com/en-us/document-viewer.html?ramdomVar=6549482870393403912&docPath=/content/dam/dcc/documents/en-us/productdatasheet/11/11-31/11-3184-sylgard-184-elastomer.pdf.

[32] Johnson K., Pashler H., Yantis S. (2002). Neural basis of haptic perception. Steven’s Handbook of Experimental Psychology.

[33] Greenspan J.D., Bolanowski S.J. (1996). The Psychophysics of Tactile Perception and its Peripheral Physiological Basis. Pain and Touch.

[34] Gescheider G.A., Bolanowski S., Hardick K. (2001). The frequency selectivity of information-processing channels in the tactile sensory system. Somatosens. Mot. Res..

[35] Hinton G.E., Salakhutdinov R.R. (2006). Reducing the Dimensionality of Data with Neural Networks. Science.

[36] Irie B., Miyake S. Capabilities of Three-layered Perceptions. Proceedings of the IEEE 1988 International Conference on Neural Networks.

[37] Cybenko G. (1989). Approximation by superpositions of a sigmoidal function. Math. Control. Signals Syst..

[38] NumPy Reference. https://numpy.org/doc/stable/reference/.

[39] Akiba T., Sano S., Yanase T., Ohta T., Koyama M. A Next-generation Hyperparameter Optimization Framework. Proceedings of the 25th ACM SIGKDD International Conference on Knowledge Discovery & Data Mining.

[40] Bergstra J., Bardnet R., Bengio Y., Kegi B. Algorithms for Hyper-Parameter Optimization. Proceedings of the Neural Information Processing Systems 2011.

[41] Keras Documentation. https://keras.io/ja/.

[42] Kingma D.P., Ba J. Adam: A Method for Stochastic Optimization. Proceedings of the International Conference on Learning Representations 2015.

[43] Ward J.R. (1963). Hierarchical grouping to optimize an objective function. J. Am. Stat. Assoc..

[44] Wishart D. (1969). An Algorithm for Hierarchical Classifications. Biometrics.

[45] SciPy User Guide. https://docs.scipy.org/doc/scipy/reference/tutorial/index.html#user-guide.

