# Supplementary material for: A Model for Estimating Tactile Sensation by Machine Learning Based on Vibration Information Obtained while Touching an Object
Source: Sensors (Basel). 2021 Nov 23;21(23):7772. doi: 10.3390/s21237772 (PMC8659637; doi:10.3390/s21237772)
Supplement: Supplementary file 1 [file sensors-21-07772-s001.zip › sensors-1420819-supplementary.pdf]

Table S1. Specific characteristics for dynamic strain amplifiers

Applicable bridge resistance	60~1000 Ω
Gauge ratio	2.00 fixed
Response frequency range	DC to 2.5 kHz (DPM-911B) or DC to 10 kHz (other amplifiers), within 10% deviation

Table S2. Specific characteristics for the static/dynamic friction measuring instrument

Measurement range	Frictional resistance: up to 9.8 N
Vertical load	Up to 500 gf
Vertical load sensitivity	0.5 gf or less
Measurement velocity	0.1~100 mm/s
Measurement distance	1~100 mm
Drive motor	AC servomotor
